# Feasibility of a telephone-delivered educational intervention for knowledge transfer of COVID-19-related information to older adults in Hong Kong: a pre–post-pilot study

**DOI:** 10.1186/s40814-022-01169-y

**Published:** 2022-10-06

**Authors:** Mong Yung Fung, Yu Hong Lee, Yan Tung Astor Lee, Mei Ling Wong, Joyce Tik Sze Li, Enoch E. Nok Ng, Vivian Wing Yan Lee

**Affiliations:** 1 Faculty of Medicine, The Chinese University of Hong Kong, Hong Kong, Hong Kong; 2 Center for Learning Enhancement and Research, The Chinese University of Hong Kong, 5/F, Hui Yeung Shing Building, Hong Kong, Hong Kong

**Keywords:** Feasibility, Telephone educational intervention, COVID-19, Elders

## Abstract

**Background:**

During the COVID-19 pandemic, educational interventions have become necessary to prevent the spread of health-related misinformation among Hong Kong older adults. The primary objective of this study was to assess the feasibility of a student-led, telephone-delivered intervention to improve COVID-19-related health knowledge among Hong Kong older adults. The secondary objective was to evaluate the impact of the intervention on the student volunteers.

**Methods:**

Twenty-five participants aged 65 or above who were able to communicate in Cantonese and had no hearing or cognitive impairments were recruited for this longitudinal pre–post-study from a community center in Hong Kong. The pilot telephone-delivered intervention consisted of five telephone call sessions conducted by 25 student volunteers. Each participant was paired with the same volunteer throughout the intervention. The first four sessions included pre-tests that assessed the participants’ understanding of three COVID-19-related themes: medication safety, healthcare voucher scheme, and COVID-19 myth-busting. Standardized explanations of the pre-test questions were offered to participants during the phone calls. In the last session, a post-test on all the themes was conducted. The intervention’s feasibility was assessed based on (a) percentage changes in the participants’ test scores, (b) attrition rate, and (c) the acceptability of the intervention by the participants. The impact of the intervention on the student volunteers was evaluated based on a student feedback survey. There was no control group.

**Results:**

Significant improvements in the participants’ test scores (out of 100%) for all themes were observed after the intervention: from 76 to 95.2% for medication safety, from 64.0 to 88.8% for the healthcare voucher scheme, and from 78.0 to 93.2% for COVID-19 myth-busting. The average improvement in test scores of the three themes was 18.4% (95% CI 12.2 to 24.6%). Most participants were satisfied with the program. The student feedback survey suggested that the intervention enhanced students’ communication skills and understanding of Hong Kong older adults.

**Conclusion:**

This pilot study offers initial evidence of the potential and feasibility of student-led, telephone-delivered educational interventions for the transfer of COVID-19-related knowledge to older adults and their benefits for the student volunteers. Future studies should include larger samples and a control group.

**Supplementary Information:**

The online version contains supplementary material available at 10.1186/s40814-022-01169-y.

## Key messages regarding the study’s feasibility

- What uncertainties existed regarding feasibility?

- It was not clear whether phone calls are a feasible means of educating older adults regarding COVID-19-related health issues.
- It was not clear whether student volunteers can benefit from delivering such a telephone-based intervention.

- What are the key findings on feasibility?

- Our pilot study offers initial evidence of the potential and feasibility of a telephone-delivered intervention for educating Hong Kong older adults and its benefits for the student volunteers.

- What are the implications of the findings on feasibility for the design of the main study?

- Telephone-based educational interventions could be adopted to transfer health-related knowledge to older adults in the future.
- Future studies should include larger samples and evaluate the potential of phone calls to improve older adults’ mental well-being during the pandemic.

## Introduction

### Background

Since early January 2020, the spread of COVID-19 has greatly affected Hong Kong. COVID-19 can be deadly, and the risk it poses to health is heightened by the spread of misinformation about the disease. As the World Health Organization stated, the spread of false information, such as fake remedies and conspiracy theories, has hampered efforts to fight COVID-19 [1].

A recent study revealed that older adults aged 65 or above were more likely to hold COVID-19-related conspiracy beliefs than younger age groups [2]. Baker et al. suggested that older age groups are usually more susceptible to health misinformation, due to memory and cognitive decline as a result of aging [3]. Another study published in 2016 found that Internet users over 65 years old shared seven times as many fake news articles as the youngest age group sampled [4]. Studies have also found that health literacy is generally lower in older age groups, especially among those with lower educational and socioeconomic levels [5, 6]. The popularization of social media has also contributed to the spread of COVID-19-related health misinformation [7]. In Hong Kong, the implementation of social distancing measures has led to the suspension of social welfare services, making it more difficult for older adults to seek advice from healthcare professionals [8] and thereby rendering them more reliant on inaccurate online information.

In Hong Kong, the well-being of older adults has been negatively affected by the spread of COVID-19-related misinformation. Without a proper understanding of COVID-19, older adults have been unable to properly protect themselves from the disease. According to one study, some older adults believed that face masks could be reused after washing them with detergent [9]. In addition, older adults may experience panic and anxiety after listening to exaggerated rumors about COVID-19 [10]. Further, frequent exposure to misinformation and conspiracy theories was found to be associated with a higher risk of depression among older adults during the COVID-19 pandemic [11].

Given the potential impact of COVID-19-related misconceptions on older adults’ health, educational interventions to clarify these misunderstandings are crucial. In addition, such interventions may provide older adults with a support system to improve their psychological well-being during the pandemic. Systematic reviews have suggested that face-to-face educational programs such as seminars and Q&A sessions are effective in improving health literacy among older adults [12, 13]. However, under social distancing policies, it is difficult to organize large-scale face-to-face health educational programs. Virtual educational programs through communication media such as mobile applications, telephone calls, and the Internet could be more feasible.

Telephone-delivered interventions may be the most appropriate means to educate older adults during the pandemic, because it is difficult to implement mobile and Web-based applications without providing prior instructions in person. Moreover, the use of mobile applications is not popular among older adults in Hong Kong, given that only 57% of them owned a smartphone in 2019 [14]. In contrast, a telephone is easier to use and more popular with older adults, suggesting that telephone-delivered educational interventions are more practical and suitable.

Studies have indicated the usefulness of telephone-delivered educational interventions for health-related issues such as smoking cessation [15] and diabetes management [16]. However, studies of telephone-delivered educational interventions for COVID-19-related health issues are lacking. Moreover, previous interventional programs have mostly been conducted by healthcare professionals. However, it could be beneficial for healthcare students from disciplines such as pharmacy, medicine, and nursing to conduct them, because studies have suggested that volunteering is an effective way to improve students’ communication with patients and colleagues [17–19]. Therefore, in the present study, we implemented a student-led, telephone-delivered intervention to educate older adults in Hong Kong about COVID-19. We expected our telephone-based educational intervention to enhance older adults’ knowledge of COVID-19.

The primary objectives of this pilot study were as follows: (a) to evaluate the feasibility of a telephone-based intervention for improving older adults’ knowledge of COVID-19, (b) to assess the acceptability of the intervention by participants, and (c) to assess the attrition rate of the intervention. The secondary objective was to evaluate the impact of the intervention on the student volunteers who implemented it.

## Methods

### Study design

This longitudinal pre–post-pilot study compared older adults’ understanding of COVID-19-related topics before and after a 5-week telephone-based educational intervention delivered by student volunteers. The participants’ knowledge of medication safety, the Hong Kong healthcare voucher scheme, and COVID-19 myth-debunking was measured through pre- and post-tests. There were no changes to the methods or outcome measurements after trial commencement. There was no control group.

### Participants

Twenty-five older adults were recruited from a local district community center for older adults in Yuen Long, Hong Kong. Participant recruitment was conducted by a social worker at the center. Those who were 65 or above, able to communicate in Cantonese, and able to communicate via phones for the telephone-delivered interventions were recruited. Those with cognitive or hearing impairments were excluded. If any participants had failed to attend all five sessions of the intervention and evaluation, they would have been excluded from the data analysis.

Twenty-five undergraduate students from the Chinese University of Hong Kong who major in biomedical sciences, medicine, pharmacy, public health, food and nutritional science, or nursing and were in their second year of study or above were recruited. All of the students were required to attend training sessions conducted by pharmacists and social workers. The training sessions covered topics relating to common geriatric diseases, medication safety, the Hong Kong healthcare system, and effective communication with older adults. The students were also required to read e-learning materials on COVID-19 and pass a quiz assessing their knowledge of medication safety, the healthcare voucher scheme, and COVID-19 before the intervention. Students who did not attend the training sessions, complete the e-learning materials, or pass the quiz were excluded from the study.

The feasibility of the intervention was evaluated based on the following criteria:

- Logistical challenges during the intervention: Older adult participants may forget or otherwise miss scheduled phone calls. Therefore, the proportion of missing data was considered.
- Potential improvement in knowledge of COVID-19-related topics: It was not clear whether a simple telephone-based intervention had the potential to improve the participants’ knowledge of COVID-19-related topics.
- Intervention acceptability and attrition rate among participants: Most older adult educational programs to date have been conducted face-to-face, before the pandemic. Also, it might be challenging for some older adults to hear on the phone due to age-related hearing loss. Therefore, it was not clear whether older adults would enjoy acquiring health-related knowledge through phone calls and complete the program. Acceptability and attrition rates among participants were thus evaluated.

Based on the above feasibility aims, specific criteria were formulated to determine progression to the larger-scale definitive trial. These included (a) proportion of missing data in the knowledge questionnaires and satisfaction surveys lower than 10%, (b) improvement in mean test scores of 10% or more, (c) mean acceptability and satisfaction levels of 80% or higher, and (d) attrition rate of 15% or below.

### Materials

#### COVID-19-related knowledge questionnaires

A pre-test questionnaire and a post-test questionnaire were designed to evaluate the participants’ knowledge of COVID-19. The pre-test questionnaire contained 20 true or false questions on three themes, each of which focused on one COVID-19-related health topic. The questions in the pre-test and post-test questionnaires were identical to facilitate accurate analysis of the impact of the intervention, because altering the post-test questions could have led to confounding. The differences in the scores between the pre-test and the post-test questionnaires were used to assess the potential of the intervention to improve the participants’ knowledge regarding COVID-19. The three health topics included in the knowledge questionnaires were relevant to the health needs of older adults in Hong Kong. The questionnaire is included in the Additional file 1.

Theme 1 contained five questions related to medication safety. During the COVID-19 pandemic, non-essential healthcare services at public hospitals in Hong Kong have been reduced. As follow-up appointments have been postponed, some chronic patients may experience a shortage of medication. Instead of consulting healthcare professionals, some of them may discontinue their medication [20]. Medication safety education is of paramount importance to ensure that older adults adhere to their prescribed medication courses during the pandemic.

Theme 2 contained five questions related to the healthcare voucher scheme. To subsidize older adults’ use of private healthcare services in Hong Kong, qualifying older adults are currently provided with healthcare vouchers worth HKD$2000 per year by the government. Although these vouchers subsidize private healthcare services only, older adults may wrongly believe that these vouchers can be used to purchase sanitizing products and face masks. Further, scams and disputes related to the healthcare vouchers have occurred occasionally during the pandemic [21]. It is therefore important to educate participants about the healthcare vouchers to reduce relevant disputes and better protect their rights.

Although the questions in themes 1 and 2 were not directly about COVID-19, they were included because the pandemic has indirectly affected other areas of healthcare. As current health advertisements focus primarily on COVID-19, educating people about other important health issues is necessary.

Theme 3 contained 10 questions that focused on the debunking of COVID-19 myths. Fake news and misinformation regarding COVID-19 are extremely prevalent, and some older adults may find it difficult to differentiate between correct and incorrect health information.

Although older adults were not involved in the design of the questionnaires, we ensured that the intervention addressed their educational needs. First, the student volunteers brainstormed and discussed the content of the questionnaires based on their previous encounters with older adults and recent publications on the needs of older adults during the pandemic. Standardized answers to these questions were formulated based on official guidelines and relevant academic publications [22–24]. Later, these questionnaires and standard answers were reviewed and checked by two registered pharmacists to ensure that they addressed the educational needs of older adults during the pandemic.

#### Participants’ feedback survey

At the end of the 5-week program, all of the participants were invited to complete a feedback survey that comprised both yes–no and open-ended questions. The feedback survey was aimed at investigating the participants’ opinions of and overall satisfaction with the intervention program. The feedback and satisfaction levels of the participants were crucial for evaluating the feasibility of the intervention. The survey is included in the Additional file 1.

##### Students’ feedback survey

To evaluate the impact of the intervention on the students who conducted it, the student volunteers were invited to respond to a survey consisting of eight open-ended questions regarding the most and least enjoyable parts of the program, major challenges encountered, new skills acquired, and preferred mode of volunteering (phone-delivered versus face-to-face). The survey is included in the Additional file 1.

##### Geriatric depression scale (GDS-15) survey

To evaluate the depression risk of the participants during the COVID-19 pandemic, the Chinese version of the Geriatric Depression Scale (GDS-15) was adopted [25]. The questionnaire contains 15 questions as a self-report assessment of the participants’ risk of depression. Out of a total score of 15, a score of 5 or above indicates a low to moderate risk of depression, whereas a score of 8 or above indicates a high risk of depression. GDS-15 was only administered in the pre-test and served as a rapid assessment of participants’ depression risk during the pandemic. Participants who scored 8 or above were referred to the social worker at the community center for follow-up action such as arranging counseling sessions.

#### Intervention and experimental procedure

The study was conducted by the recruited students under the supervision of two registered pharmacists in Hong Kong. Oral consent from the study participants and the students was obtained prior to the intervention. Each participant was paired with one student throughout the intervention. One theme was covered per week from weeks 1 to 4. The students were required to write a summary of the phone call after each intervention session. The overall flow of the study is summarized in Table 1.

**Table 1:** Overview logistic plan

Time	Tasks
Before week 1	• Training session on intervention protocol and communication techniques
Week 1	• Self-introduction • GDS-15 survey • Pre-test and explanations on theme 1: medication safety
Week 2	• Pre-test and explanations on theme 2: health care voucher scheme
Week 3	• Pre-test and explanations on theme 3: COVID-19 myth-debunking (first 5 questions)
Week 4	• Pre-test and explanations on theme 3: COVID-19 myth-debunking (later 5 questions)
Week 5	• Posttest on theme 1, 2, and 3 • Subject feedback survey
After week 5	• Student feedback survey

During week 1, the students first introduced themselves and provided the participants with an overview of the telephone-delivered educational program. The participants were asked to provide their preferred time for conducting the phone calls. The GDS-15 survey was also administered to determine the mental well-being of the participants.

During weeks 1 to 4, the pre-test questionnaire covering the three aforementioned health topics was administered at the start of each phone call session to measure the participants’ knowledge of the health topics before intervention. All of the true or false questions were asked verbally via phone by the students. The participants’ answers were then marked by the students. One mark was awarded if the participant answered a question correctly and was also able to justify their answer correctly. Marks were not awarded for wrong answers or answers without reasonable justifications. The total scores for the pre-test and post-test questionnaires were 20 marks.

The intervention was designed to maximize the learning efficiency of the participants. When a participant answered a pre-test question incorrectly, the student provided the participant with the correct answer and a standardized explanation. The provision of rapid feedback enabled the participants to understand their knowledge gaps and the volunteers to better correct the participants’ misconceptions [26, 27]. The participants were also invited to raise other health-related questions during the phone calls, allowing the participants to obtain personalized feedback from the students and engage with the learning materials more effectively. The 40 questions were spread over the four sessions to avoid overwhelming the participants. As studies have suggested that attention span tends to decrease with age [28, 29], spreading the educational content over five sessions was expected to optimize learning efficiency [30]. Each phone call was expected to last for 10 min on average.

Several measures were taken to reduce potential variation in intervention delivery by different students. First, the students were provided with standardized answers to the questions. Further, prior to the program, the students were required to attend training sessions organized by pharmacists and social workers, in which the intervention protocol and tips for improving communication with older adults were introduced. In addition, the students were reminded not to give the participants any hints about the answers to ensure fairness. This ensured that the participants answered the questionnaires based primarily on their own knowledge. Further, the students were instructed to only contact the participants during the five scheduled intervention sessions.

The post-test questionnaire, which comprised 20 questions covering the three health themes, was administered in week 5. Unlike the pre-test questionnaire, correct answers and explanations were not given when the participant answered incorrectly, because the post-test questionnaire was aimed at evaluating changes in the knowledge of the participants and was not part of the intervention itself. Written information on the questions, answers, and explanations was sent to the social worker of the center; the participants could collect this information after the fifth call.

#### Sample size

As the aim of this pilot study was to assess feasibility, formal sample size calculation was not performed. However, based on the statistical guidelines proposed by Julious [31] and by Kieser and Wassmer [32], a sample size of 25 was considered appropriate and sufficient to provide reasonable estimations of the variance and effect size, which will be useful in formal sample size calculations for future studies. In another paper, Cocks and Torgerson estimated that the sample size required in a pilot study should be approximately 9% of that of the main study [33]. Because we aimed to conduct the main study with roughly 250 participants, 25 participants were sufficient for the present pilot study to obtain the evidence required to progress to the main study. In addition, to ensure that the study was conducted promptly during the COVID-19 pandemic and to reduce the time required for participant recruitment, a sample size of 25 was considered a reasonable recruitment target.

#### Data processing and statistical analysis

The feasibility criteria, based on the primary outcomes, were improvement in knowledge of COVID-19-related health topics, attrition rate, proportion of missing data in surveys, and intervention acceptability among the participants. Improvement in knowledge was measured by the differences between the pre-test and post-test scores for the three themes with a 95% confidence interval (CI). The mean, standard deviation (SD), and range of the test scores were also considered. Intervention acceptability was assessed based on the participants’ satisfaction with the intervention, as obtained from the participants’ feedback survey. Descriptive statistics were calculated to analyze the results for yes–no questions in the feedback survey, while qualitative analysis was conducted for open-ended questions. The impact of the intervention on the student volunteers was measured based on a qualitative analysis of the results of the students’ feedback survey. The data collected were recorded in Microsoft Excel and SPSS Version 25.0 was used for the statistical analysis.

## Results

Twenty-five older adults from the aforementioned community center were screened and recruited. The phone calls were made between October and November 2020. All of the participants completed the study, resulting in a 0% attrition rate. There were no missing data in any of the questionnaires and surveys. The sociodemographic characteristics of the participants at baseline are summarized in Table 2.

**Table 2:** Sociodemographic characteristics of participants at baseline

Age (mean ± SD) and gender (*N* (%))	
Mean age (years old)	71.7 ± 4.8
Male	9 (36%)
Female	16 (64%)
Educational qualifications (*N* (%))	
Have received primary education or above	25 (100%)
Have received secondary education or above	14 (56%)
Physical health (*N* (%))	
With chronic diseases	15 (60%)
Hypertension	7 (28%)
Hypercholesterolemia	3 (12%)
Diabetes	2 (8%)
Other chronic diseases	3 (12%)
Without any chronic diseases	10 (40%)
GDS-15 score (mean±SD) and classification (*N* (%))	
Mean GDS-15 score	3.84 ± 3.1
Below 5 marks (no risk of depression)	16 (64%)
5 marks or above (low to moderate risk of depression)	9 (36%)
Average phone call duration (minutes)	23.7±14.6

### Change in knowledge questionnaire scores

The average questionnaire scores increased from 74.0 (SD: ± 15.0%) before the intervention to 92.5 (SD: ± 8.43%) after the intervention, which translates into an absolute increase of 18.4% (95% CI 12.2 to 24.6%) in average test scores. Significant improvements in the questionnaire scores for all three themes were observed: from 76.0 (SD: ± 30.5%) to 95.3 (SD: ± 8.72%) for medication safety, from 64.0 (SD: ± 31.6%) to 88.9 (SD: ± 20.1%) on the healthcare voucher scheme, and from 78.0 (SD: ± 15.4%) to 93.0 (SD: ± 8.52%) for COVID-19 myth-debunking. The absolute increase in the test scores for the three themes was 19.2% (95% CI 7.1 to 31.3%) for medication safety, 24.8% (95% CI 10.3 to 39.3%) for the healthcare voucher scheme, and 37.6% (95% CI 24.5 to 50.7%) for COVID-19 myth-debunking.

### Content of phone conversations

Apart from answering the knowledge questionnaires and receiving information on health-related issues, 24 of the participants (96%) actively shared information on their personal lives during the five phone calls. In the first phone call, five participants (20%) expressed their concerns about issues such as insufficient face mask supplies, social isolation, and personal medical conditions. In the second call, 15 participants (60%) described how they normally used the vouchers provided by the healthcare voucher scheme. In the third and the fourth calls, 10 participants (40%) shared the measures that they adopted to protect themselves from the virus, such as wearing face masks and washing hands, as well as the channels from which they usually sought health-related information. Although the participants were encouraged to ask any health-related questions, only half of them did so (50%) during the phone call sessions.

### Acceptability of telephone-delivered intervention

Overall, the intervention was well accepted, as reflected by the high satisfaction level among the participants. Our satisfaction survey showed that almost all of the participants agreed that the duration of the phone calls (96%) and the difficulty of the questions (88%) were appropriate. Only three participants (12%) found the questions to be too easy. Further, many of the participants (96%) agreed that the program was useful and had greatly enhanced their understanding of COVID-19. In addition to knowledge enhancement, many (92%) of the participants agreed that they felt more cared for and more hopeful after the program:

It’s nice that I got to talk to young people via this program. [Participant 6]

I felt happier and learned a lot after the program. [Participant 13]

It’s nice and heartwarming that I got to talk to the same person during every phone call session. [Participant 17]

Although the telephone-delivered approach excluded face-to-face interaction, all of the participants agreed that the telephone-based intervention was flexible and convenient (100%). Eighteen of the participants (72%) preferred telephone-based over face-to-face programs, as they felt that phone calls are more convenient and personalized. A few (28%) of the participants preferred face-to-face programs, because they offer more authentic and genuine interactions.

When asked about the shortcomings of the program, around four (16%) of the participants wished that face-to-face seminars had been incorporated into the intervention. Another three (12%) would have preferred to have been notified of the phone call schedule earlier so that they could have better arranged their plans. The remaining 17 (72%) did not think that the program had any significant deficiencies.

### Impact of telephone-delivered intervention on students

All 25 student volunteers completed the program. Their demographic characteristics at baseline are summarized in Table 3. Overall, most of the students (92%) enjoyed the program, the major reasons for which included the flexibility (56%), meaningfulness (16%), and appropriate duration (32%) of the program. Only two students (8%) did not find any parts of the program to be enjoyable.

**Table 3:** Demographic characteristics of student volunteers

Prior experiences in telephone-based volunteering	
Yes	10 (40%)
No	15 (60%)
Gender	
Male	6 (24%)
Female	19 (76%)
Academic profile	
Mean year of study	2.76±1
Year 2	15 (60%)
Year 3	1 (4%)
Year 4	9 (36%)
Majors	
Biomedical sciences	2 (8%)
Medicine	9 (36%)
Pharmacy	7 (28%)
Public health	3 (12%)
Food and nutritional science	1 (4%)
Nursing	3 (12%)

Most of the students believed that they were able to apply skills taught in class in the program. Twelve students (48%) believed that they were able to apply the medical knowledge that they had learned in class. Another four students (16%) mentioned using other skills/knowledge, such as communication skills and bioethics knowledge. The remaining 11 students (44%) did not think that they were able to apply any of the skills acquired at school during the program.

Regarding the major obstacles encountered during the phone call sessions, many of the students (46%) found it difficult to communicate with the participants solely via phone calls. Another five students (20%) had difficulties in contacting the participants, and three students (12%) found it difficult to sustain their conversations with the participants. Five students (20%) did not encounter any difficulties throughout the program.

When asked about the major benefits of the program, 23 students (92%) believed that their communication skills had improved significantly. Although the students found it difficult to bond with the participants in the absence of face-to-face interactions, 10 students (40%) believed that they had become more sensitive to and cautious about verbal cues during the intervention. In addition, 19 students (76%) believed that the program had deepened their understanding of older adults in Hong Kong, as the older adults were more knowledgeable than they had expected. Also, the students appreciated the opportunity to meet and collaborate with teammates from other health-related disciplines. Twenty-four (96%) students communicated with their teammates regarding issues such as the logistics of making phone calls, educational content, and questions raised by the participants.

During the design of the COVID-19 knowledge questionnaires, students from different healthcare disciplines were responsible for setting different questions. For instance, pharmacy and public health students helped to design questions on medication safety and the healthcare voucher scheme, respectively. In addition, the questionnaires were cross-checked by the students before the program.

Furthermore, interprofessional engagement benefited the participants by improving the quality of the intervention delivered. For example, when public health students were asked about medication problems by the participants during the phone calls, they were able to seek advice from the pharmacy students.

Despite the benefits associated with the program, only seven students (28%) preferred telephone-based interventions to face-to-face interventions, while 17 (68%) liked face-to-face interventions more. The remaining student did not have any strong preferences.

## Discussion

### Potential of intervention to improve participants’ knowledge of COVID-19

As evidenced by the improvement in the knowledge questionnaire scores, the telephone-delivered educational intervention has the potential to enhance participants’ knowledge of COVID-19-related health issues. A similar pilot study found that a telephone-delivered educational intervention was effective in improving the knowledge of older adults during the Hong Kong SARS epidemic in 2007 [34]. In contrast with the present study, only two phone calls, instead of five, were conducted in the abovementioned study to avoid information overload. Nonetheless, our findings suggest that increasing the quantity of content delivered by dividing it across more phone call sessions might not affect the improvement in test scores.

Multiple factors might have contributed to the significant improvement in the participants’ test scores. First, the educational content delivered in the program was easy to understand, as most of the participants deemed the difficulty level appropriate. Second, the educational program was personalized and able to address various questions raised by each participant, because our phone calls were conducted on a one-on-one basis.

However, our findings might not be applicable to other population groups. A meta-analysis published in 2014 analyzed five randomized controlled trials and concluded that phone call interventions were not more effective than standard care for the glycemic control of diabetes patients [35]. The studies reviewed were mostly conducted in rural areas, where diabetic patients typically have lower economic and education levels, leading to poorer medication adherence [35, 36]. Therefore, the significant improvement in the test scores observed in the present study may be attributable to the fact that Hong Kong is a well-developed city and all of our participants had received at least primary education.

Nonetheless, this does not imply that phone call interventions are not useful in rural areas; in fact, phone-based interventions may be particularly useful in remote areas because health education in these regions is limited [37]. However, the content of interventions should be adjusted based on based on participants’ education and knowledge to maximize outcomes.

The improvement in the test scores was higher for questions related to COVID-19 than for questions on the other topics. This could have occurred partly because people’s knowledge of COVID-19 has generally improved as the pandemic has progressed, owing to extensive media coverage and public health campaigns [38, 39]. The participants performed less well for other health topics, including the healthcare voucher scheme and medication safety, potentially because these health topics have received less emphasis in health campaigns during the pandemic.

### Intervention acceptability

The participants were highly satisfied with the phone-based intervention, due to its usefulness and ability to improve their moods. Research has shown that many older adults feel isolated due to social distancing policies [40], and the results of the Geriatric Depression Scale (GDS-15) survey suggested that nine of the participants (36%) were at risk of developing depression. Meanwhile, the results of the participant satisfaction survey showed that some of them (46%) felt more cared for and more positive after the program. Our results suggest that even without any rigorous training for volunteers, phone-based interventions have the potential to improve participants’ psychological well-being during the pandemic. A similar study conducted recently also found that lay-person-delivered phone calls reduced the loneliness and depression of the participants during the pandemic [41].

### Impact on students

In addition to its positive impact on the participants, the telephone-based intervention benefited the students who delivered it. First, the students were able to practice their phone-based communication skills and use of verbal cues. Although face-to-face patient communication has been heavily emphasized in health-related disciplines, phone-based communication skills are equally important in scenarios such as patient referral, telephone consultation services, and breaking bad news [42, 43]. Therefore, the telephone communication skills acquired through the present study are important for students of health-related disciplines in their future practice.

Second, the program improved the students’ understanding of older adults in Hong Kong. They realized that older adults in Hong Kong are more knowledgeable than they had expected. Although the students’ attitudes toward older adults were not formally assessed in the present study, misconceptions of and prejudices against older adults are prevalent among health professionals and students of health-related disciplines [44]. When students with negative attitudes towards older adults become health professionals, they are more likely to offer poor patient care [45]. The fact that the students in the present study found the older adults to be more knowledgeable than they expected suggests that their perceptions of older adults became more positive. This finding suggests that volunteering work with older adults has the potential to improve students’ attitudes toward older adults.

Moreover, interdisciplinary collaboration between the students benefited both the students and the participants in the program. Such collaboration enabled students from their majors to learn from each other and share their expertise. For instance, the questionnaires were cross-checked by the students before the program. Likewise, when public health students were asked about medication problems by the participants during the phone calls, they were able to seek advice from the pharmacy students. Our results suggest that such interdisciplinary collaboration helped to ensure information accuracy during phone calls, consistent with previous studies [46, 47].

### Satisfaction level of students

Although most of the students enjoyed the program, due to its flexibility and appropriate duration, only half of them (56%) were able to apply the medical knowledge they had learned at university during the intervention. Some students were able to apply medical knowledge from their university studies to answer additional health-related questions asked by the participants, such as questions about the safety of vaccinations and the side effects of certain drugs. As these questions were not included in the standardized set of answers, the students who were paired with these participants had to answer their inquiries based on what they had learned at university. However, some participants asked no such additional questions. In these cases, the students were not able to apply what they had learned, as they only needed to rely on the standardized answers.

Although most of our student volunteers had limited experience of interacting with older adults, some of them found it more challenging to communicate with the participants than others. This occurred possibly because the quality of communication was dependent on many factors, such as the communication style and personalities of the participants and the students [48]. Hence, some students found it easier to deal with the participants assigned to them than others did.

Nevertheless, our results suggest that the majority of the students found it challenging to interact and sustain their conversations with the participants. Studies have suggested that telecommunication education is lacking in both medical [49] and nursing training [50]. To address this issue, workshops to train student volunteers on telephone-based communication are important. In addition, students should be trained in delivering educational content via video-conferencing, which allows for better interactions between the students and participants while retaining the flexibility of telephonic interventions.

### Progression to future definitive trials

As all of the progression criteria were satisfied in this pilot study, future definitive trials will be planned. As some student volunteers found it difficult to contact the participants, it is suggested that in future trials student volunteers send message reminders to participants one day prior to each scheduled phone call.

Second, video conferencing could be incorporated into future interventions. The satisfaction survey suggested that some participants who felt socially isolated wished for face-to-face interventions to be incorporated for more genuine and natural interactions. However, face-to-face activities cannot be conducted when social distancing policies are enforced. Video-conferencing could be a feasible substitute for face-to-face interactions to enhance the authenticity of communication and the well-being of older adults in Hong Kong [51] without increasing the risk of COVID-19 infection. Moreover, video-conferencing has been found to be well accepted within Hong Kong older adult populations [52].

### Implications: Telehealth in Hong Kong

The study offers preliminary evidence that the implementation of telehealth in Hong Kong is feasible, owing to the high acceptability of the intervention. “Telehealth” refers to the provision of health services remotely, via digital communication media such as phone calls, video conferencing, and the short messaging services (SMS) [53]. During the pandemic, many medical appointments and patient education seminars have been canceled. Such issues could be addressed through the use of telehealth. Although studies in the USA [54] and Canada [55] have shown that telehealth is feasible and able to supplement traditional care, no similar studies of the potential of telehealth have been conducted in Hong Kong. In the absence of direct evidence, the present study’s finding that phone calls were well accepted by the older adult participants for delivering health education provides indirect evidence of the potential of telehealth in the Hong Kong context.

### Limitations

This study has several limitations. We were unable to measure effect sizes and conduct significance tests as statistical testing was generally inappropriate in feasibility studies. Including a control group would have been difficult, as community centers typically want all of their participants to receive the intervention. A waitlist control study design might be a plausible alternative.

Another potential limitation is that the improvement in the participants’ knowledge of the health-related topics might not be long-lasting, owing to the short study period. Follow-up phone calls could be useful to measure knowledge retention among participants after the intervention.

Moreover, the sample was small and the participants were recruited non-randomly and from only one district community center. Around 56% of the participants had received secondary education, whereas the average for Hong Kong older adults is 39.6% [56]. Therefore, our findings might not be applicable to older adults in Hong Kong with different demographic characteristics. To address this issue, the investigators of the present study have commenced a similar but larger-scale program with a sample size of 300. This ongoing study may offer more insights into the feasibility and impact of the program.

Finally, no pre- or post-questionnaires were administered to measure the impact of the intervention on the participants’ depression level and mood. Future studies should include relevant surveys to quantify the impact of their interventions on participants’ mental well-being.

## Conclusion

In conclusion, our pilot study offers preliminary evidence that telephone-based interventions for educating Hong Kong older adults on COVID-19-related issues are feasible. The study also revealed that the intervention improved the student volunteers’ communication skills and understanding about older adults in Hong Kong.

## Supplementary Information

**Additional file 1.**

## Data Availability

The datasets used and/or analyzed during the current study are available from the corresponding author on reasonable request.
