# Supplementary material for: Feasibility of a telephone-delivered educational intervention for knowledge transfer of COVID-19-related information to older adults in Hong Kong: a pre–post-pilot study
Source: Pilot Feasibility Stud. 2022 Oct 6;8:228. doi: 10.1186/s40814-022-01169-y (PMC9535844; doi:10.1186/s40814-022-01169-y)
Supplement: Supplementary file 1 — Additional file 1. [file 40814_2022_1169_MOESM1_ESM.docx]

**Additional file 1**

1. COVID-19 related knowledge questionnaires (True/False questions)

*Theme 1 (Medication Safety)*

1. It is appropriate to stop a course of antibiotics early when symptoms disappear (False)
2. If you find it difficult to swallow tablets, it is appropriate to crush it. (False)
3. When medications prescribed by the doctor failed to manage your conditions well, you should take additional herbal or Chinese medications to alleviate your symptoms (False)
4. It is unnecessary to consult your doctor or pharmacist if you experience any side effects such as vomiting and headaches after taking prescribed medications (False)
5. In case of drug shortage during the pandemic, it is harmless to borrow drugs with similar therapeutic effects from your neighbors. (False)

*Theme 2 (Health Care Voucher)*

1. The accumulation limit of the health care voucher is HKD $5000. (False)
2. Health care vouchers can be used to purchase facemasks at pharmacy. (False)
3. Health care vouchers can be used to pay for COVID-19 testing fee at private hospitals. (True)
4. Health care vouchers can be used to purchase medications at pharmacy. (False)
5. Health care vouchers can be used for payment of accommodation fee at public hospitals. (False)

*Theme 3 (COVID-19 myth debunking)*

1. Face masks can be worn inside out. (False)
2. COVID-19 is airborne. (False)
3. COVID-19 can be prevented and treated by antibiotics. (False)
4. COVID-19 can be prevented by pneumococcal vaccine. (False)
5. Medical masks easily accessible at pharmacy are protective enough against COVID-19. (True)
6. Face masks can be sterilized and reused after heating. (False)
7. One should wash their hands after removing one’s masks. (True)
8. All health information on social media platforms is accurate. (False)
9. Only elderly but not younger adults are at risk of getting COVID-19. (False)
10. Surgical masks should not be worn more than 8 hours. (True)
11. Subject’s feedback survey

| Questions | Answers |
| --- | --- |
| Multiple Choice questions | |
| 1. Is the phone call duration appropriate? | - Yes, it is appropriate. - No, it is too short. - No, it is too long |
| 1. Are the questions in knowledge surveys of appropriate difficulty? | - Yes, they are of appropriate difficulty. - No, they are too difficult. - No, they are too easy |
| 1. Did the intervention improve your understanding towards COVID-19? | - Yes, it did. - No, it did not. |
| 1. Did you become more hopeful after the intervention? | - Yes, I did. - No, I did not. |
| 1. Do you want to join similar telephone-based programs in the future? Why? | - Yes, I do. - No, I do not. |
| 1. Do you agree that telephone-based programs are convenient? | - Yes, I do. - No, I do not. |
| 1. Do you prefer face-to-face or telephone-based programs? Why? | - I prefer face-to-face programs - I prefer telephone-based programs. - I do not have specific preferences. |
| 1. Overall, do you find this program helpful? | - Yes, I do. - No, I do not. |
| Open-ended questions | |
| 1. What do you like about the program? | N/A |
| 1. Do you have any suggestions on how to improve the program? | N/A |

1. Students’ feedback survey (Open-ended questions)
2. What do you like about the educational program?
3. What do you dislike about the educational program?
4. What have you learnt from the program? Did you manage to develop any new skills?
5. Were you able to apply any skills and/or knowledge learnt during your undergraduate studies to this program?
6. After completing the program, is there anything that is very different from your expectation?
7. Do you have any suggestions on how to improve the project?
8. Do you prefer telephone-based or face-to-face volunteering? Why?
9. What were your major challenges during the program? How did you overcome them?
10. Geriatric Depression Scale (GDS-15) survey (Yes/No questions)
11. Are you basically satisfied with your life during the pandemic?
12. Have you dropped many of your activities and interests during the pandemic?
13. Do you feel that your life is empty during the pandemic?
14. Do you often get border during the pandemic?
15. Are you in good spirits most of the time during the pandemic?
16. Are you afraid that something bad is going to happen to you during the pandemic?
17. Do you feel happy most of the time during the pandemic?
18. Do you often feel helpless during the pandemic?
19. Do you prefer to stay at home, rather than going out and doing new things during the pandemic?
20. Do you feel you have more problems with memory than most during the pandemic?
21. Do you think it is wonderful to be alive now during the pandemic?
22. Do you feel pretty worthless the way you are now during the pandemic?
23. Do you feel full of energy during the pandemic?
24. Do you feel that your situation is hopeless during the pandemic?
25. Do you think that most people are better off than you are during the pandemic?
